# Notes and Comments

**Published:** 1896-05

**Authors:** 


LOUIS JACK.

### Notes and Comments.[1]

THE USE OF GOLD SOLDER SCRAPS.—Dr. R. E. Sparks, in the *Dominion Dental Journal*, says, "Dealers cannot allow more than two-thirds as much for solder scraps as they charge for, say, eighteen-carat solder. In crown- and bridge-work solder scraps may be made as valuable as new solder.

"Clean all old backings and bits of plate having solder upon them by dropping them into a bath of dilute sulphuric acid and boiling for a few minutes, or leave in the bath overnight. Wash them in clean water. Hammer each piece quite thin and cut into small pieces, and put into a bottle or box for use. They answer every purpose where cusps are to be filled, or any place where bulk of solder is required. To use them, first flow some new solder upon the piece, then add the scrap. The application of heat will flow the solder contained in the scrap and fuse the whole mass together. Should the surface be rough, on account of the insufficiency of the solder contained in the scrap, to cover the bits of plate contained in the same, add more solder."

THE DENTIST'S POSITION.—We quite agree with Dr. Welch where he says, "A dignified bearing is respected, but too much familiarity is contemptible; genuine sympathy is endearing, but officious intermeddling is resented.

[1] The assistant editor solicits contributions for this department,—new methods, new remedies and formulas, or any short practical note which may prove of value to the practitioner or student. Address 1718 Walnut Street, Philadelphia.

"It requires greater wisdom to know our place and keep it, before our patients, than to preside at a public gathering; it shows greater discretion to say the right thing at the right time, in the right way, under ordinary circumstances, than to be eloquent before an enthusiastic audience; it is better to do the common duties of the hour well than to do some great thing out of time and out of place. It should therefore be our ambition to be in our proper place, doing our work with faithfulness and fidelity."

---

Removal of Blood-Spots.—Dr. J. E. Woodward says that the removal of blood-spots on the clothing can be promptly accomplished by the application of pyrozone.

---

Finishing Cement Fillings.—"Instead of paraffin, which scales off as soon as wet, melt together rosin and wax on a spatula, and pour on cement filling after it has stood a few minutes. After a day or two it will take a polish about like ivory."—Dr. E. T. Darby.

---

Trichloracetic Acid in Cleaning Teeth.—A two-per-cent. aqueous solution of trichloracetic acid to moisten pumice, Dr. W. H. Jones writes, is perfectly harmless. He states further that he has used it in his practice for some time, and finds it far superior to iodine tincture for removing the "green stain" on children's teeth.

---

Fundamental Requisites to Secure Professional Success.—In speaking of the management of dental practice, Dr. Louis Jack very truly says that without the combination of professional training, general intelligence, moral rectitude, courtesy of manner, and personal neatness, there is little probability of any one securing a practice which would require methodical control. These elements are all so essential that at any stage of practice the loss of any of the several may impair the career of any one.

---

A Hint to Dental Students.—In the dental college class, it is safe to predict that the successful man in after life will be the student who is studious, courteous, and gentlemanly. Those who are only desirous of obtaining their degree, who are ill behaved and unkempt, whether in the lecture-room or infirmary, will be the ones who will bewail the lack of patronage, and find that they do not fill the measure necessary to success.—*Western Dental Journal.*

---

Religion of the Body.—Mr. Andrew Clark, the eminent physician, in a recent lecture at Birmingham on the "Religion of the Body," in which he spoke of the body as a talent which must be put to the highest possible uses, and emphasized the necessity of obedience to the laws of physical health, used the phrase "physical righteousness,"—a capital phrase, which ought to be written on the minds of all Americans. Physical righteousness means obedience to the laws of health; means, among other things, exercise, rest, and the avoidance of overwork. There are many persons who are morally righteous and physically unrighteous. This kind of unrighteousness has been one of our national sins.—*The Outlook.*

---

The Open Mind.—A recent writer in *The Outlook* says that one of the most discouraging features in a political campaign is the fact that no sooner do most men begin to discuss politics than they betray such absolute rigidity of conviction that discussion is profitless.

This tendency is not confined to political questions. Open-mindedness is one of the very highest and rarest qualities in every walk of life. Nothing is more erroneous than the impression that in order to be strong one must be a partisan in everything that concerns his life. Open-mindedness is a willingness to learn from events; not to accept as a finality any statement of a great question, or position on a discussion. Truth, of course, is eternal, but the expressions of truth in declaration and in institutions are always changing, and he who would live by the truth must keep himself open to its new revelations. No fate is sadder than to become stationary in a world which is moving on into new and growing times and demanding growing men.

